# Outcomes of Upper Gastrointestinal Bleeding Based on Time to Endoscopy: A Retrospective Study

**DOI:** 10.7759/cureus.7325

**Published:** 2020-03-19

**Authors:** Sheikh A Saleem, Pujitha Kudaravalli, Sana Riaz, Venkata Satish Pendela, Dongliang Wang, Dhruv Lowe, Divey Manocha

**Affiliations:** 1 Gastroenterology, State University of New York (SUNY) Upstate Medical University, Syracuse, USA; 2 Internal Medicine, State University of New York (SUNY) Upstate Medical University, Syracuse, USA; 3 Internal Medicine, Rochester General Hospital, Rochester, USA; 4 Public Health and Preventive Medicine, State University of New York (SUNY) Upstate Medical University, Syracuse, USA

**Keywords:** endoscopy, upper gastrointestinal bleeding, timing, interventions, mortality

## Abstract

Introduction

Non-variceal upper gastrointestinal bleeding (UGIB) is a major burden on the health care system. The timing of endoscopy has been an ongoing debate and data on the association of early endoscopy with a better or worse clinical outcome are conflicting. In our study, we aimed to identify the benefits versus the risks of performing an urgent endoscopy in regards to the number of endoscopic interventions, length of hospital stay, number of packed red blood cells (PRBCs) transfused, and mortality.

Methodology

This is a retrospective record-based study. A total of 806 charts were reviewed and 251 patients with the signs and symptoms of UGIB on presentation were included in the study. Patients with variceal bleeding, lower gastrointestinal bleeding, insignificant bleeds with no drop in H/H, GI bleed not being the presenting complaint on admission, and patients on anticoagulation were excluded.

Results

Out of the patients who underwent an urgent esophagogastroduodenoscopy (EGD), 26.2% needed a second-look EGD 48 hours after the first EGD when compared to 4% and 2% in the early (12-24 hours) and late (>24 hours) endoscopy groups, respectively. In patients who underwent urgent EGD, 23% had active bleeding and it was statistically significant when compared to the other groups. The active bleeding limited the visualization during the endoscopy, which led to a repeat EGD in the urgent EGD group. If an endoscopic intervention was received, patients having EGD >24 hours received a smaller number of interventions. There was no statistical difference in the Blatchford scores between the three groups, indicating that the groups were similar in morbidity. No difference in mortality, hospital length of stay, or number of blood transfusions received, surgical or interventional radiology-guided interventions was found between the three groups.

Conclusion

Patients who underwent urgent endoscopy had more procedures, with no difference in mortality, number of units of blood transfused, or length of hospitalization when compared to the early or late endoscopy groups.

## Introduction

Non-variceal upper gastrointestinal bleeding (UGIB) is a major burden on the health care system and accounts for 300,000 hospitalizations in the United States alone over one year [1]. Thirty-six per 100,000 patients present with UGIB with a male to female ratio of 2:1 and a mean age of 52. Mortality associated with UGIB is decreasing with advancements in endoscopy, but the costs associated with the in-hospital management of UGIB has been on the rise, with an approximate expenditure of 7.6 billion dollars in 2009 [2-3].

The most common risk factors for non-variceal UGIB are the overuse of nonsteroidal anti-inflammatory medications (NSAIDs), Helicobacter pylori infection, the use of antiplatelet and anticoagulation medications, aspirin, and selective serotonin reuptake inhibitors. On presentation, two large-bore intravenous cannulas are secured, and fluid resuscitation is started immediately in UGIB. Proton pump inhibitor (PPI) infusion is also started although intermittent PPI therapy is comparable to bolus plus continuous PPI infusion [4]. Blood products are used when the hemoglobin falls to less than 7 g/dL and vasopressor therapy is started when there is hemodynamic instability despite fluid resuscitation.

Endoscopy identifies the cause of bleeding in 80% of cases and remains the cornerstone of diagnosis and therapy in GI bleeding. The timing of endoscopy has been an ongoing debate and the data on the association of early endoscopy with better or worse clinical outcomes are conflicting. The timing of endoscopy is also influenced by the weekend phenomenon where patients admitted over the weekend tend to undergo endoscopy later due to the unavailability of resources. The latest National Institute for Health and Care Excellence (NICE) guidelines recommend endoscopy of unstable patients with severe UGIB immediately after resuscitation and to all other patients with UGIB within 24 hours of admission. However, clinical evidence in relation to the timing of endoscopy in stable patients is very low in accordance with the Grading of Recommendations Assessment, Development and Evaluation (GRADE) criteria and there is very little literature on unstable patients. The American Society of Gastrointestinal Endoscopy (ASGE) differs in that they define urgent endoscopy as within 24 hours of admission and recommend adequate resuscitation and proton pump inhibitor therapy before endoscopy [5-6].

In a retrospective study by Yarovski et al., comorbid illness is the primary cause of death in UGIB and not the bleeding itself [2]. This further supports that resuscitating the patient and hemodynamic stability precede over the timing of endoscopy to improve mortality. Several studies have been conducted to evaluate the timing of endoscopy. In a systematic review done by Kelvin et al. and a retrospective study conducted by Alexandrino G et al., early endoscopy within 12 hours did not reduce the re-bleeding rate or improve survival [7-8]. Clinical trials to evaluate the outcomes based on the timing of endoscopy are sparse, as it is considered unethical to delay endoscopy when a patient might require it.

In our study, we aimed to identify the benefits versus the risks of performing an urgent endoscopy in regards to the number of endoscopic interventions, length of hospital stay, number of packed red blood cells (PRBCs) transfused, and mortality.

## Materials and methods

Data collection

The study involved a chart review of patients who got upper GI endoscopy done from 01/01/2017 to 12/31/2017 at Upstate Hospital for acute upper GI bleeding. Filter criteria of upper gastrointestinal bleeding and dates from 01/01/2017 to 12/31/2017 were used to extract the list of patients with a GI bleed. Out of the 806 charts reviewed, patients with variceal bleeding, lower gastrointestinal bleeding, insignificant bleeds with no drop in H/H and stable vitals, GI bleed not being the presenting complaint on admission, patients on anticoagulation were excluded from the study. A total of 251 patients were included in the study, which included patients presenting to the hospital with signs and symptoms of UGIB. Endoscopy reports were reviewed and patients with upper GI bleeding were included. Blatchford scores were calculated for all patients. Time to endoscopy, site of bleed, endoscopic interventions, number of units of blood transfused, length of hospitalization, inpatient mortality, interventional radiology (IR), or surgical interventions were collected. The primary endpoints of the study were to evaluate if the timing of endoscopy had an influence on the number of endoscopic interventions, bleeding during the procedure obscuring visualization, and requiring a repeat EGD. The secondary endpoints were to evaluate if there was any difference in the hospital length of stay, inpatient mortality, the number of units of blood received, and surgical/IR interventions in regard to the time to endoscopy.

Definitions

Urgent endoscopy is defined as endoscopy within 12 hours of admission. Early endoscopy is defined as endoscopy between 12 and 24 hours of admission. Late endoscopy is when endoscopy is performed more than 24 hours after admission.

Statistical analysis

This is a retrospective observational study and the statistical analysis was exploratory in nature. For descriptive purposes, continuous variables were summarized by the number of non-missing values, mean and standard deviation, median, minimum, maximum, and discrete variables were summarized by frequency and proportion. One-way analysis of variance (ANOVA) was used to compare the differences between groups for continuous variables and chi-square tests were used for discrete variables. Fisher exact tests were used as the nonparametric alternative if more than 50% of expected cell counts were less than five. All statistical analyses were performed using SAS 9.4 (SAS Institute, Cary, North Carolina) with a significance level of 0.05 for any hypothesis testing.

## Results

A total of 806 charts with gastrointestinal bleeding were reviewed, out of which 251 included. Twenty-four (24) patients had upper gastrointestinal bleeding secondary to bleeding varices (9%) and they were excluded. Patients were divided into three groups: those that received esophagogastroduodenoscopy (EGD) in <12 hours of admission (urgent endoscopy), 12-24 hours of admission (early endoscopy), and >24 hours after admission (late endoscopy). The baseline demographics of the patients, including age, sex and Blatchford score, are shown in Table 1. There was no difference in age, sex, and Blatchford scores between the three groups. 

**Table 1:** Baseline demographics 37.70% of patients were under the age of 50 in the urgent EGD group when compared to 24.47% and 30.21% in the early and late EGD groups. 54.26% of patients were between the age groups of 51 and 70 in the early EGD group when compared to 44.26% of patients in the urgent EGD and 38.54% in the late EGD groups. 18.03%, 21.28%, and 31.25% of patients were between the age groups of 71-89 in the urgent, early and late EGD groups, respectively. The patients did not differ in age between the three groups. 52.46% were males and 47.54% were females in the urgent EGD group, 60.64% were males and 39.36% were females in the early EGD group, and 65.63% were males and 34.38% were females in the late EGD group. Gender did not differ between the urgent, early, and late EGD groups with a p-value of 0.258. Blatchford score was similar amongst the three groups, with a p-value of 0.992 and a mean of 8.30, 8.49 and 8.72 and a median of 8, 9 and 9 in the urgent, early, and late EGD groups, respectively. EGD: esophagogastroduodenoscopy

Age	Urgent EGD	Early EGD	Late EGD	p-value
<50yrs	23 (37.70%)	23 (24.47%)	29 (30.21%)	0.091
51-70yrs	27 (44.26%)	51 (54.26%)	37 (38.54%)	
71-89yrs	11 (18.03%)	20 (21.28%)	30 (31.25%)	
Sex				
Male	32 (52.46%)	57 (60.64%)	63 (65.63%)	0.258
Female	29 (47.54%)	37 (39.36%)	33 (34.38%)	
Blatchford score				
Number	61	92	96	0.992
Mean (SD)	8.30	8.49	8.72	
Median	8	9	9	
Minimum Blatchford score, Maximum Blatchford score	0,17	0,17	0,18	

A significant association was noted between the need for a second look and the timing of EGD with p<0.001, as shown in Table 2. Of the patients who underwent an urgent EGD, 26.2% needed a second-look EGD 48 hours after the first EGD. For patients who underwent EGD, 12-24 hours and more than 24 hours after admission, the proportions of patients needing a second-look EGD was only 4% and 2%, respectively. A statistically significant association was also noted between the timing of EGD and the blood obscuring the scope with a p-value of 0.007, as seen in Table 2. In patients who underwent urgent EGD, 23% of patients had active bleeding. This could have obscured visualization and treatment of the bleeding site. For patients who underwent early EGD and late EGD, the proportion of patients who had active bleeding was only 16% and 10%, respectively.

**Table 2:** Need for second-look endoscopy and blood obscuring visualization during the first scope EGD: esophagogastroduodenoscopy

	Urgent EGD	Early EGD	Late EGD	p-value
Need for second-look EGD				.001
Yes	16 (26.23%)	4 (4.26%)	2 (2.08%)	
No	45 (73.77%)	90 (95.74%)	94 (97.92%)	
Blood on EGD				0.007^
Active bleeding	14 (22.95%)	15 (15.96%)	10 (10.42%)	
Signs of recent bleed	13 (21.31%)	17 (18.09%)	7 (7.29%)	
no bleeding	34 (55.74%)	62 (65.96%)	79 (82.29%)	

There was no difference in the need for IR-guided procedures and surgery between the three groups, as seen in Table 3.

**Table 3:** Interventional radiology or surgical interventions when endoscopy fails to control bleeding 91.80%, 93.62%, and 95.83% of patients did not require any additional procedures in the urgent, early, and late EGD groups, respectively. 4.92%, 2.13%, and 1.04% required IR and 3.28%, 2.13%, and 1.04% required surgical interventions in the urgent, early, and late EGD groups, respectively. EGD: esophagogastroduodenoscopy

Non-variceal bleeding				
Interventional radiology (IR) or surgical interventions	Urgent EGD	Early EGD	Late EGD	
No additional procedures	56 (91.80%)	88 (93.62%)	92 (95.83%)	0.595
IR	3 (4.92%)	2 (2.13%)	1 (1.04%)	
Surgery	2 (3.28%)	2 (2.13%)	1 (1.04%)	
Both	0 (0.00%)	2 (2.13%)	2 (2.08%)	

Endoscopic interventions like clips, cautery, argon plasma coagulation, and epinephrine are used to stop the bleeding. Among patients with non-variceal bleeding, 47 out of 61 (47+14) patients receiving EGD within 12 hours did not receive any intervention, five patients received one intervention, and nine patients received more than one intervention. Whether the intervention was received was not related to the time of EGD. However, if the intervention was received, patients having EGD >24 hours received fewer numbers of interventions, as shown in Table 4.

**Table 4:** Endoscopic interventions during endoscopy 35.71%, 33.33%, and 77.78% received one endoscopic intervention in the urgent, early, and late EGD groups, respectively. 2+ interventions were done in 64.29%, 66.67%, and 22.22% in urgent, early, and late EGD, respectively. There was no statistically significant difference between the 1 and 2+ intervention. 77.05%, 77.66%, and 81.25% received no endoscopic intervention in the urgent, early, and late EGD groups, respectively. 22.95%, 22.34%, and 18.75% received more than one intervention in urgent, early, and late EGD, respectively. There was no statistically significant difference between the no intervention and 1+ intervention. However, if the intervention was received, patients having EGD >24 hours received a less number of interventions. EGD: esophagogastroduodenoscopy

	Urgent EGD	Early EGD	Late EGD	p-value
Number of interventions				
1 intervention	5 (35.71%)	7 (33.33%)	14 (77.78%)	0.011
2+ intervention	9 (64.29%)	14 (66.67%)	4 (22.22%)	
				P value
No intervention	47 (77.05%)	73 (77.66%)	78 (81.25%)	0.767
1+ intervention	14 (22.95%)	21 (22.34%)	18 (18.75%)	

No differences in length of hospital stay, number of units of blood, and mortality were noted in association with the timing of EGD, as seen in Table 5.

**Table 5:** Mortality, length of hospitalization, and blood transfusion 1.64%, 1.06%, and 1.04% mortality was seen in urgent, early, and late EGD groups and there was no statistically significant difference between the three groups. 54.24%, 54.88%, and 54.43% patients stayed in the hospital for 3-4 days, 23.73%, 24.39%, and 25.32% stayed in the hospital between 5-7 days, and 22.03%, 20.73%, and 20.25% patients stayed in the hospital for >7 days in the urgent, early, and late EGD groups. EGD: esophagogastroduodenoscopy; PRBC: packed red blood cell 54.10%, 48.94%, and 50% patients received one or more blood transfusion in the early, urgent, and late endoscopy groups.

Mortality	Urgent EGD	Early EGD	Late EGD	p-value
Yes	1 (1.64%)	1 (1.06%)	1 (1.04%)	1.000*
No	60 (98.36%)	93 (98.94%)	95 (98.96%)	
Length of hospitalization				0.999
3-4 days	32 (54.24%)	45 (54.88%)	43 (54.43%)	
5-7days	14 (23.73%)	20 (24.39%)	20 (25.32%)	
>7days	13 (22.03%)	17 (20.73%)	16 (20.25%)	
Blood transfusion				
Number of PRBC=0	28 (45.90%)	48 (51.06%)	48 (50.00%)	0.812
Number of PRBC>0	33 (54.10%)	46 (48.94%)	48 (50.00%)	

## Discussion

Upper gastrointestinal bleeding is a gastrointestinal emergency with a high mortality rate (10%) whilst posing a significant economic burden on the health care system. It is important to risk stratify patients early in the course of presentation to improve mortality and morbidity and to reduce health care costs. Several scoring systems have been used to determine the disposition of a patient and risk for rebleeding/complication. Patients with low Blatchford scores that is 1 or less do not need to be admitted to the hospital and can be safely discharged without an inpatient endoscopy [9-12]. The management of low-risk UGIB outpatients reduces costs, as expenditure is primarily due to inpatient hospitalizations ($13,059 for the UGI-bleed cohort vs. $729 for the general population cohort) [13-14].

Fluid resuscitation, appropriate blood transfusion, and antacid therapy are the first steps in the management of UGIB and should be initiated immediately. Pharmacotherapy with antacids is shown to reduce re-bleeding, size of the culprit lesion, and surgery but has not shown to reduce all-cause mortality [9,15]. Current recommendations include a loading dose of antacid followed by a continuous infusion starting on admission and for 72 hours after endoscopy, subsequently transitioning it to oral therapy. A study by Worden et al. has shown that intermittent PPI therapy after endoscopy in higher-risk stigmata and oral PPI therapy in lower-risk patients are equally effective when compared to continuous PPI infusion in treating UGIB and this will help in reducing significant costs. Contrarily, a budget impact analysis done by Lu et al. showed that the incremental costs of using different PPI regimens (continuous versus intermittent, duration) are modest (~$200) when compared to the total in-patient cost [16-17].

Endoscopy, the next step in management, continues to play a pivotal role in the diagnosis and treatment of nonvariceal upper GI bleeding [18-19]. Time to endoscopy continues to be a topic of debate because of the disparate recommendations suggesting endoscopy at different time intervals. The timing of endoscopy is also influenced by the weekend effect, which refers to an increase in adverse outcomes of patients admitted on a weekend. The factors contributing to the weekend effect are not having an adequate number of specialists on call and disproportionate staffing to patient load ratio causing a delay in the time to endoscopy when compared to a weekday admission. Several studies have shown the prevalence of weekend effect in the United States and adverse outcomes related to it although no cause-effect relationship has been established [20]. Contrary to this hypothesis, an audit conducted in the UK showed that the time to endoscopy was independent of the weekend/weekday effect or whether the endoscopy was performed in the endoscopy suite versus the operating room [21]. International consensus on the management of patients with nonvariceal upper gastrointestinal bleeding recommends early endoscopy that is within 24 hours for most upper gastrointestinal bleeding.

The Forrest classification is used to classify the lesions seen on endoscopy. Clean-based ulcers are low risk for re-bleeding, and these patients can be discharged on the same day with a pharmacological agent and no endoscopic therapeutic intervention is needed. Ulcers actively bleeding/spurting and ulcers with a visible vessel and a clot on the top are high risk for bleeding and endoscopic intervention is recommended. Mechanical, thermal, injection, and topical endoscopic interventions are available to stop the bleeding and these patients are observed for at least 72 hours after the endoscopy [22]. Esophagogastroduodenoscopy, when performed too early without adequate resuscitation, could potentially lead to endoscopic interventions. In our study, we observed that patients who underwent urgent endoscopy needed more endoscopic interventions like clips, cautery, epinephrine, and argon plasma coagulation when compared to patients who underwent late endoscopy. Patients who underwent endoscopy later in the course likely received pharmacological therapy with PPI and adequate resuscitation, which could have ceased the bleeding and reduced the size of the culprit lesion. In addition, approximately 80% of the upper GI bleeds stop spontaneously and the amount of blood in the stomach decreases overtime. There is also a progression of the bleeding ulcer into a clean-based ulcer and this happens over 72 hours, leading to a smaller number of endoscopic interventions in late endoscopic patients [23-24].

We also observed that the patients who underwent urgent endoscopy tend to have a second-look EGD. The active bleeding during an urgent EGD obscures the scope, limiting visualization and hindering the diagnosis and therapy of the culprit lesion. Gastric lavage is commonly done prior to an EGD to enhance visualization. Several studies have also shown that the use of erythromycin, which accelerates gastric emptying prior to endoscopy improves visualization but the studies differ in secondary outcomes, including the need for second-look endoscopy, length of hospital stay, number of units of blood transfused, and mortality. A few studies have compared gastric lavage with a nasogastric tube prior to endoscopy versus erythromycin and found no difference between the two and hence erythromycin can be used without the insertion of a nasogastric tube, which is considered painful and uncomfortable. Although erythromycin is shown to be beneficial, the studies also differ in the dosage, the ideal timing of the medication before endoscopy, and duration. More studies are needed to understand the clinically useful endpoints [25-26].

Mortality, number of units of blood transfused, and length of hospitalization were not influenced by the timing of endoscopy in our study population. This is consistent with a study done by Sarin N et al., in which no statistical difference was found in mortality, need for surgery, or blood transfusion between groups receiving endoscopy within six hours of presentation and between six and 24 hours of presentation [27]. Upper GI bleeding is a marker of deterioration of diagnosed and undiagnosed co-morbidities rather than a cause of death and physicians should be vigilant and treat the co-morbidities beyond the bleeding episode. The mortality seen in a UGIB is predicted by the overall physical status of the patient [28]. In a study done by Sung et al., it was shown that 80% of death in UGIB is not related to bleeding and is caused by pulmonary disease, cardiac disease, malignancy, and multiorgan failure. Patients with GI bleed-related mortality succumbed in the first three days and those with hemodynamic shock, active bleeding on index endoscopy, nonsteroidal anti-inflammatory drugs (NSAIDs), and aspirin users were at a higher risk. The location of bleed (gastric, duodenum, anastomotic) had no bearing on mortality. Hence, the optimization of comorbidities and cardiopulmonary resuscitation should be given more importance over treating the bleeding lesion in the acute setting [29].

Studies have conflicting results on the relation between time to endoscopy and mortality in high-risk patients. The three groups in our study had patients with a similar risk based on Blatchford scores and no difference in mortality and length of hospitalization was noted. In a study by Kumar NL et al., it was shown that low-risk patients who underwent urgent endoscopy that is within 12 hours of presentation had a higher chance of inpatient re-bleeding, need for surgical or radiological intervention, and endoscopic re-intervention. It was also observed that the time to endoscopy did not influence the outcome in high-risk patients [3]. Contrarily, it was observed that the high-risk patients with bleeding had a lower mortality rate when they underwent an early endoscopy that is within 12 hours of presentation. In a clinical trial conducted by Lin et al., patients with clear or coffee-ground nasogastric aspirate did not benefit from EGD within 12 hours and patients with a bloody nasogastric aspirate had benefited from an early procedure. These results are probably skewed, as they only included bleeding from peptic ulcer disease and had a potential outlier effect [30].

In addition, we found no difference in the number of interventions, second-look EGD, mortality rate, and hospital length of stay between patients who had early and late endoscopy. Some studies have shown that even if a patient did not undergo EGD when the bleeding stopped with antacid therapy and resuscitation, the long-term and short-term outcomes were similar to those that did undergo EGD. This is contrary to a study by Garg et al., where higher mortality was observed in patients who received EGD more than 24 hours of presentation when compared to those undergoing the procedure within 24 hours. The mortality was highest in groups that did not undergo EGD at all. Hospital length of stay was shorter in early EGD patients in their study [20].

Our study is limited by the presence of unmeasured biases given the retrospective nature of the study. Data on the use of erythromycin before endoscopy were also not collected, which could have affected the visualization in each group. Nevertheless, risk factors and outcomes are measured and can provide useful information to apply in clinical practice. Randomized controlled trials are ethically challenging in upper GI bleeding as a patient cannot be delayed treatment if appropriate.

## Conclusions

Patients who underwent urgent endoscopy, that is, within 12 hours of admission had a higher chance of undergoing repeat EGD within 48 hours likely secondary to blood obscuring visualization during the first EGD. In addition, patients in the urgent EGD group had more procedures with no difference in mortality, the number of units of blood transfused or the length of hospitalization when compared to the early (12-24 hours) or late (>24 hours) endoscopy group. No difference was found between the early and late endoscopy groups in any of the above-mentioned outcomes. Based on our study, performing an endoscopy earlier on leads to more interventions, putting the patient at a higher risk for procedure-related complications and adverse events due to a lack of sufficient resuscitation. Through our study, we would like to emphasize that importance has to be given to the optimization of comorbidities, cardiopulmonary resuscitation, and pharmacological therapy over treating the bleeding lesion with endoscopy in the acute setting. Endoscopy can be safely performed when the patient is resuscitated and stabilized.
